# The Effect of Bovine Milk on the Growth of *Bombyx mori*

**DOI:** 10.1673/031.013.9801

**Published:** 2013-09-30

**Authors:** Niharika Konala, Praveena Abburi, Venugopal Reddy Bovilla, Anitha Mamillapalli

**Affiliations:** Department of Biotechnology, GITAM Institute of Science, GITAM University, Visakhapatnam- 530 045, India.; # These authors contributed equally to this work

**Keywords:** economic traits, growth rate, mulberry leaves, silkworms

## Abstract

*Bombyx mori* L. (Lepidoptera: Bombycidae) is a well-studied Lepidopteran model system because of its morphology, life cycle, and economic importance. Many scientists have placed importance on enhancing the economic traits of *B. mori* because it's larvae, silkworms, are vital in the production of silk. In this study, the effect of bovine milk on *B. mori* growth was tested. Bovine milk contains several components that aid in healthy growth. The treatment was given to fifth instar *B. mori* larvae because the fifth instar period is when *B. mori* eats voraciously and shows maximum growth among all its larval stages. The larvae were treated with fresh mulberry, *Morus* L. (Rosales: Moraceae), leaves and mulberry leaves dipped in milk from the first day of the fifth instar. Treatments were given on alternate days, and the silkworms were weighed every day to determine whether milk had any role in enhancing the weight of the larvae. Cocoon weights were measured, as the weight indicates the approximate amount of silk that can be reeled. The results showed that larvae gained 82.5% more weight by the end of fifth instar larval when fed with mulberry leaves dipped in milk than when fed with fresh mulberry leaves without milk. The larvae fed with milk-treated leaves gained 310% weight from day 1 to day 7 of the fifth instar, while the larvae fed with fresh leaves gained 153% weight in the same timespan. In addition, cocoon weight increased by 8% when milk was added compared to when it was not. These results suggest that *B. mori* larvae can be fed mulberry leaves treated with bovine milk for better growth rate and increased silk production.

## Introduction

Silkworms are larvae of the moth *Bombyx mori* L. (Lepidoptera: Bombycidae), which is native to Asia and spins a cocoon of fiber that is the source of commercial silk. *B. mori* feeds on the leaves of the mulberry tree, *Morus* L. (Rosales: Moraceae), and serves as an excellent model system because of its life cycle, it is inexpensive, and there are no ethical issues involved (Hiroshi et al. 2008). Many people in India are involved in sericulture, which involves a great deal of labor and intensive care. In order to make sericulture more economically viable, it has become important to study and analyze various factors that improve growth, yield, fiber quality, and larvae's resistance to pathogens.

Enriching mulberry leaves by nutrient supplementation is one of the ways to improve growth rate in *B. mori*. The effect of mulberry leaves enriched with amino acids on the growth of *B. mori* has been studied (Khan and Saha 1995; Nirwani and Kaliwal 1998; Radjabi 2010; ). The protein content of the silk gland, fat body, and muscles was found to increase significantly when larvae were fed with ascorbic acid (Quraiza et al. 2008). Different combinations of mineral nutrients were found to improve larval growth and silk production (Ahmad 1993). Mulberry leaves enriched with nickel chloride and/or potassium iodide have increased cocoon weight at low concentrations (Islam et al. 2004). The growth and development of larvae, and subsequent cocoon production, are greatly influenced by the nutritional quality of mulberry leaves (Masthan et al. 2011).

Artificial diets rich in amino acids are required for optimal growth of an insect, and casein has been widely used as it contains all amino acids (Panizzi and Parra 1991). Casein contains fatty acids, cholesterol, sugars, vitamins, and minerals (Vanderzant 1966). The presence of casein in a diet has enhanced the growth rate of *Manduca sexta* caterpillars and was observed to stimulate the feeding efficiency of *B. mori* (Ito 1960; Woods 1999). Diet supplements containing fatty acids and carbohydrates were shown to have a regulatory effect on fatty acid synthesis in larval stages of *B. mori* (Horie and Nakasone 1970). Sterols including cholesterol had a positive influence on dietary efficiency in *B. mori* (Ito et al. 1963).

Milk is a complex liquid that simultaneously provides bioactive compounds in the form of proteins, carbohydrates, fatty acids, minerals, and other nutrients that facilitate healthy growth and development. It contains peptides, polyamines, and enzymes. Many bioactive proteins and peptides derived from milk are potential modulators of various regulatory processes. The physiological significance of these substances is not yet fully understood, but both the mineral binding and cytomodulatory peptides derived from bovine milk are now claimed to be health-enhancing components (Haug et al. 2007). One such bioactive compound identified in human and bovine milk is transforming growth factor (TGFβ), which helps in differentiation, development, and immune response (Hughes et al. 2000).

Considering the beneficial properties of milk, the importance of protein, carbohydrates, and lipids in the insect diet, and the economic importance of *B. mori*, whether bovine milk has any impact on increasing the economic traits of silkworm was studied. The results showed that larval weight of fifth instar *B. mori* larvae fed with mulberry leaves dipped in bovine milk increased 3 times compared to the larvae fed with fresh mulberry leaves not dipped in milk. Milk also showed a positive effect on cocoon weight.

The rural economy of many states in India depends on sericulture, and any knowledge to improve the current state of sericulture is of immense importance. The present study gives promising results to the sericulture farmers.

## Materials and Methods

### Silkworms

*B. mori* (BV CSR2 × CSR4) were taken from the Department of Sericulture, Government of Andhra Pradesh. The larvae were reared in cardboard boxes at 23 ± 1° C and 65–70% RH. They were fed with fresh mulberry leaves until the fifth instar stage.

### Composition of Milk

Ultra high temperature processed toned milk manufactured by Visakha Dairy (local dairy farm) was used. The nutrition facts per 100 mL were as follows: 3.0 g milk fat, 4.9 g carbohydrate, 3.6 g proteins, 0.07 g minerals, 1.8 g saturated fat, 0.010 g cholesterol, 175 IU added vitamin A, 120 IU added vitamin D, 62.0 K calories energy value, 28.0 K calories energy from fat.

### Experimental Design

The experiment was designed to determine if milk had a positive effect on the growth of *B. mori*. Milk was given to fifth instar larvae, the fifth instar being the most important stage for silk production.

The mulberry leaves were dipped in milk and air dried before giving them as feed to the silk larvae, and no mortality or frass was observed. Paraffin sheets were laid, and the larvae were reared in cardboard boxes. The fecal matter was removed to provide hygienic conditions for healthy growth.

For the purpose of comparative analysis, the larvae were divided into 2 groups. Each group contained 20 larvae. Group 1 was fed with fresh mulberry leaves throughout the fifth instar and was considered as the control group. Group 2 was fed with mulberry leaves dipped in milk on alternate days (days 1, 3, and 7) of the fifth instar. On the other days (days 2, 4, and 6), larvae were fed with fresh mulberry leaves. The weights of the larvae in the 2 groups were recorded daily from day 1–7 of the fifth instar until cocoon formation. Cocoon weights were also recorded, as cocoon weight is an important parameter in determining the amount of silk that can be produced.

## Results

### Effects of fresh mulberry leaves and mulberry leaves dipped in milk on larval weight

The possibility of using milk as a growth promoter in silkworms was tested in the present study. Silkworms underwent fresh mulberry leaves and mulberry leaves dipped in milk treatments as described in the experimental design. The treatment was carried from day 1 through day 7 of the fifth instar, after which the worms started spinning and cocoons were formed. Weights were recorded from day 1 through day 7, and the average values were used for comparative analysis.

**Figure 1.**
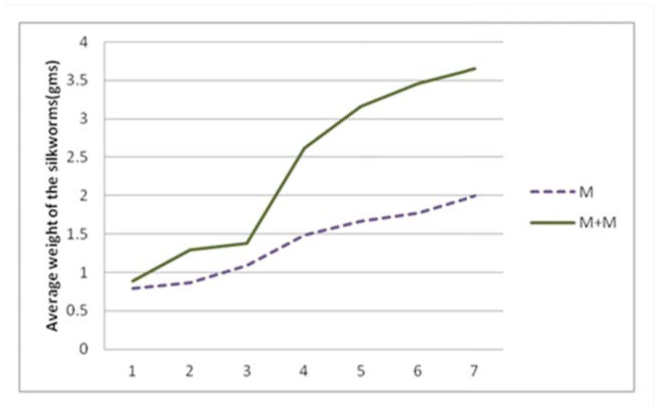
Relationship between weight of the larvae during different days of the fifth instar when fed with fresh mulberry (M) and mulberry leaves dipped in milk (M+M). Milk treated leaves were provided to the larvae on alternate days (days 1, 3, 5, and 7). The X-axis in the graph indicates the days of the fifth instar. The Y-axis indicates the average weight of 20 larvae in each group. High quality figures are available online.

Larvae that fed on the leaves treated with milk showed moderate weight gain after the first treatment (day 1). The second treatment (day 3) resulted in an exponential increase in weight from day 4. The trend continued till day 7 (Figure 1). After day 7, the weights stabilized. The reason the weights stabilized could be because the larvae reached spinning stage and stopped eating leaves after day 7.

**Figure 2.**
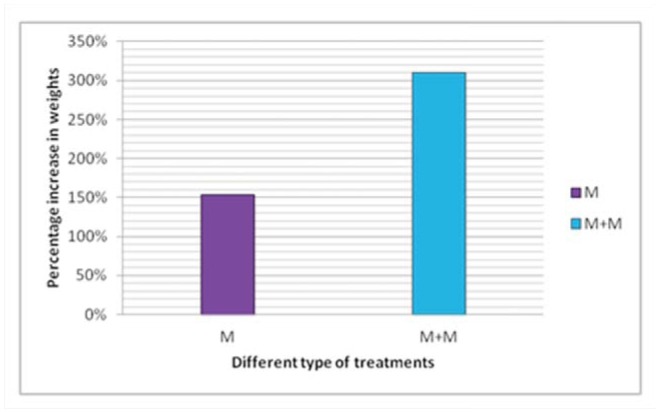
The larvae fed with milk treated leaves (M+M) gained 310% weight from day 1 to day 7 of the fifth instar, while the larvae fed with fresh leaves (M) gained 153% weight during th same period. The X-axis in the graph indicates the two treatments. The Y-axis indicates the percent increase in the body weights of the larvae. High quality figures are available online.

By the end of the fifth instar, larvae gained 82.5% more weight when fed with mulberry leaves dipped in milk compared to silkworms fed with fresh mulberry leaves. The larvae fed with milk treated leaves gained 310% weight from day 1 to 7, while the larvae fed with fresh leaves gained 153% weight in the same span (Figure 2). The results showed that milk had a positive effect on the growth of *B. mori* larvae.

### The effects of the treatments on cocoon weight

**Figure 3.**
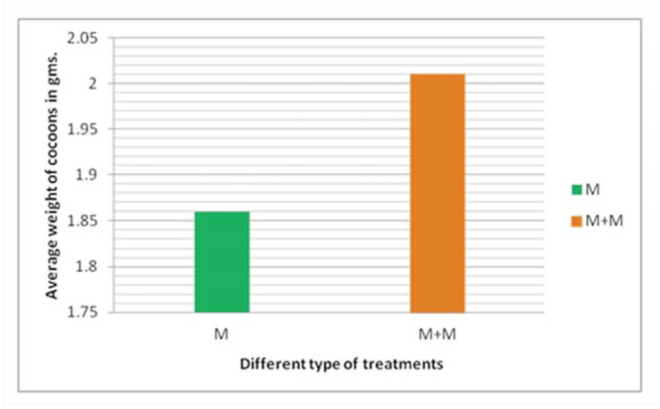
The relationship between the weights of the cocoons at the end of the fifth instar when larvae were fed with fresh mulberry (M) and mulberry leaves dipped in milk (M+M). The X-axis in the graph indicates the different treatments. The Y-axis indicates the average weight of the 20 cocoons. High quality figures are available online.

Cocoons that are sold in the market place are preferred by weight, as cocoon weight forecasts the approximate quantity of silk that can be reeled. The weight of the cocoon decreases gradually as moisture evaporates from the body of the pupa and the stored fat is consumed during the metamorphosis process (Lee 2005). The results showed that cocoons formed by worms treated with mulberry leaves dipped in milk weighed more than those formed by worms fed with fresh mulberry leaves (Figure 3). Cocoon weight increased by 8% after milk treatment when compared to control worms.

## Discussion

*B. mori* has been used for domestication because of the economic value of silk. It is also a good model system because it is large, is easy to rear, has a low cost, is not involved in ethical issues, does not present a biohazard danger. A successful cocoon crop in sericulture depends mostly on healthy larval growth (Islam et al. 2004).

In the last few years, great importance has been given to the enhancement of nutrition *B. mori* larvae. Numerous studies have reported arepertoire of compounds for improving the economic traits of the larvae, such as body weight and cocoon weight (Bentea et al. 2011). It was observed that nickel chloride can be used at low concentrations for enhancing the economic traits of *B. mori* larvae (Islam et al. 2004). Aspargine- and alanine-enriched diets for larvae did not result in a significant increase in silk production (Radjabi 2010). When fed with 1% and 2% ascorbic acid, significant increases in the protein content of the silk gland, fat body, and muscles were observed (Quraiza et al. 2008). Lactose is the main carbohydrate component in milk, and *B. mori* was shown to have a beta--glucosidase enzyme that is active on cellobiose and lactose (Byeon 2005). The presence of this enzyme suggests that *B. mori* larvae can be fed with milk, as lactose does not pose any problem for digestion. The major protein component of milk is casein, which was shown to be beneficial for *B. mori* development (Ito 1960). Casein contains a high amount of glutamic acid, and *B. mori* larvae need glutamic and aspartic acid for proper growth (Ito and Arai 1965). The effect of different vitamins on the nutritional enrichment of mulberry leaves was examined by Kanafi (2007), and it was found that all the vitamins showed a positive effect on *B. mori* growth and development. Though some of the individual components of milk were checked previously for their growth enhancement activity on *B. mori*, the combination of these components present naturally in milk was not checked. Our study showed that bovine milk had a positive effect on the body weight and cocoon weight of *B. mori* larvae.

The results of our study agree with earlier studies of bovine milk and growth (Loh et al. 1999; Black et al. 2002). The larvae may have gained weight because milk is nutrient-rich, but the exact cause of weight-gain needs to be investigated in further experiments. These results suggest that *B. mori* larvae can be fed with bovine milk treated mulberry leaves for increased growth rate and silk production.
